# The Effect of pH and Ion Channel Modulators on Human Placental Arteries

**DOI:** 10.1371/journal.pone.0114405

**Published:** 2014-12-09

**Authors:** Tayyba Y Ali, Fiona Broughton Pipkin, Raheela N Khan

**Affiliations:** Division of Medical Sciences & Graduate Entry Medicine, School of Medicine, University of Nottingham, Royal Derby Hospital Centre, Uttoxeter Road, Derby DE22 3DT, United Kingdom; University of Southampton, United Kingdom

## Abstract

Chorionic plate arteries (CPA) are located at the maternofetal interface where they are able to respond to local metabolic changes. Unlike many other types of vasculature, the placenta lacks nervous control and requires autoregulation for controlling blood flow. The placental circulation, which is of low-resistance, may become hypoxic easily leading to fetal acidosis and fetal distress however the role of the ion channels in these circumstances is not well-understood. Active potassium channel conductances that are subject to local physicochemical modulation may serve as pathways through which such signals are transduced. The aim of this study was to investigate the modulation of CPA by pH and the channels implicated in these responses using wire myography. CPA were isolated from healthy placentae and pre-contracted with U46619 before testing the effects of extracellular pH using 1 M lactic acid over the pH range 7.4 - 6.4 in the presence of a variety of ion channel modulators. A change from pH 7.4 to 7.2 produced a 29±3% (n = 9) relaxation of CPA which increased to 61±4% at the lowest pH of 6.4. In vessels isolated from placentae of women with pre-eclampsia (n = 6), pH responses were attenuated. L-methionine increased the relaxation to 67±7% (n = 6; p<0.001) at pH 6.4. Similarly the TASK 1/3 blocker zinc chloride (1 mM) gave a maximum relaxation of 72±5% (n = 8; p<0.01) which compared with the relaxation produced by the TREK-1 opener riluzole (75±5%; n = 6). Several other modulators induced no significant changes in vascular responses. Our study confirmed expression of several ion channel subtypes in CPA with our results indicating that extracellular pH within the physiological range has an important role in controlling vasodilatation in the human term placenta.

## Introduction

Throughout pregnancy, it is essential for the placenta to manage acid-base balance within a narrow pH range in order to minimise adverse effects on fetal growth and development. This is accomplished by eliminating acids formed by normal fetal and placental metabolism via the maternal circulation and through buffering provided principally by hemoglobin and bicarbonate [1], [2]. In addition, during labor, uterine contractions can occlude blood flow hence placental perfusion by compression of the uterine artery [3]. This can lead to fetal hypoxia and acidosis thus monitoring of umbilical cord blood pH is a useful measure of fetal wellbeing, indicating the need for clinical intervention when pH poses a danger to fetal health [2].

The fetoplacental circulation consists of arteries and veins of the umbilical cord, chorionic plate and stem villi. Non-innervated, chorionic plate arteries (CPA) and veins branch directly from the umbilical cord over the fetal surface of the placenta, lying closest to the fetus. Fetoplacental arteries are also much less sensitive to vasoactive molecules that have potent effects in other vascular beds [4], [5] however the effects of molecules and factors produced locally that have the capacity to alter placental vascular function have not been widely studied yet may highlight mechanisms by which control of fetoplacental vascular tone is achieved.

The placental circulation shares many similarities with the pulmonary circulation including vasoconstriction in response to hypoxia (hypoxic pulmonary vasoconstriction, HPV) [6]. The placental counterpart to this phenomenon, hypoxic fetoplacental vasoconstriction (HFPV) is mediated by small (<500 µM diameter) fetoplacental arteries [7]. Hypoxia can in turn lead to the accumulation of H^+^ ions and alter the pH of the cell's microenvironment [7]. In contrast, using *ex vivo* placental cotyledons instead of isolated blood vessels or cells, altering the pH with or without altering the pO_2_ of the perfusate had little effect on HFPV [8], perfusion pressure or the pressor response [9]. In some pregnancies, inadequate or shallow invasion of the maternal spiral arteries at the uteroplacental interface leads to poor vascular perfusion of the placenta which is observed in pre-eclampsia and is seen in some forms of intra-uterine growth restriction [10]. Moreover acidosis is associated with intrauterine growth-restricted pregnancies indicating detrimental outcomes with chronic hypoxia.

A plethora of ion channels has been described in controlling the tone of vascular smooth muscle (VSM). Depolarisation of the myocyte membrane results in opening of L-type voltage-gated calcium (Ca^2+^) channels (LTCC) that elevate cytosolic Ca^2+^ to drive vasoconstriction. Relaxation involves interplay of several channels with calcium-activated potassium (K^+^) channels having key roles. Of these, the large -conductance calcium-activated K^+^ channel, BK_Ca_ (MaxiK) enjoys widespread distribution in many vascular beds [11], [12] while channels of small (SK_Ca_) and intermediate (IK_Ca_) conductance are primarily localised to the endothelium and are targets of endothelium-derived hyperpolarising factors [13]. Calcium-activated and voltage-gated K^+^ (Kv) channels are expressed in the human placenta with a role for (Kv) channels proposed in the placental response to HFPV where the Kv blocker 4-aminopyridine (4-AP) mimicked this effect [7]. While good evidence exists for functional expression for the aforementioned channels in CPA [14], [15], there are less data for two-pore potassium (K2P) channels in the placenta. These channels first described in yeast [16], [17], play a role in determining resting membrane potential and generate leak currents [18]. Intriguingly, they are subject to local regulation by physical and chemical factors in the vicinity that include pH, oxygen tension and lipids [18]. K2P channels are distinguished from other K^+^ channels by the presence of four transmembrane segments circumscribing two pore forming domains [18]. For example, TWIK and TWIK-related K^+^ (TREK) demonstrate high sensitivity to acidic pH inside the cell while the activity of the TWIK related acid-sensitive K^+^ (TASK) is inhibited by weak changes in extracellular pH [17], [19], [20]. TASK channels open at rest maintain the resting membrane potential and are inhibited by extracellular acidic pH which makes two of the main subtypes TASK-1 and TASK-3, key candidates for responding to pH stress within the placenta. Earlier work has identified the expression of TASK-1 and TREK-1 in placental cytotrophoblast with little evidence of their functional role. Ion channel expression and function in the fetoplacental vasculature is likely to exhibit a similar repertoire of channels found in most vascular beds that differ in their functional input to the control of vessel tone. The aim of this study was to identify the ion channels in resistance-sized CPA arteries, in particular the channel subtypes mediating the effects of low extracellular pH.

## Materials and Methods

Ethical approval for the study was obtained from the Derbyshire Research Ethics Committee (04/Q2401/13) with patients providing fully informed, written consent prior to elective lower segment Caesarean section (ELLSCS) surgery. Whole placentae and myometrial biopsies were obtained from term (≥37 wks), non-laboring, singleton pregnancies following delivery at the Royal Derby Hospital and transported to the laboratory within twenty minutes. Reasons for ELLSCS included maternal request, previous ELLSCS or breech presentation. Patients with diabetes (pre-existing or gestational), high blood pressure, a history of cardiac or renal disease, or patients receiving pharmacological treatment other than iron supplementation and smokers were excluded from the study. Pre-eclamptic patients were recruited based on criteria of hypertension (>140/90 mm Hg) on two separate occasions, more than 4 hrs apart after 20th week of gestation in previously normotensive woman and proteinuria (0.3g/L over 24 hours [21]. Table 1 shows the demographics of patients recruited.

**Table 1:** Patient demographics for normal and pre-eclamptic (pregnancies). Data are presented as mean (± SEM) or median [± IQR]. * p<0.05.

Parameter		NORMAL	PE	p
		N = 40	N = 6	ns: not significant
**Maternal age (yrs)**		31.25±0.9	29±2.3	0.3 ns
**Primipara n (%)**		11 (27.5%)	4 (66%)	-
**Prepregnancy Body mass index (kg/m^2^)**		25 [22]–[32]	26.5 [23]–[30]	0.9 ns
**Max. systolic blood pressure outside labour (mmHg)**		125±3.2	150±6.1	0.01 *
**Max. diastolic blood pressure outside labour (mmHg)**		63±1.5	96±4	0.003 *
**Proteinuria g/L**		-	3.97±0.7	-
**Gestational age at delivery (days)**		271±1.2	236±8.0	0.0009 *
**Birth weight (g)**		3242±73	2128±320	0.0003 *
**Birth weight centile**		48 [15–73]	30 [18]–[50]	0.4 ns
**Placenta/Birth weight ratio**		0.2±0.02	0.3±0.09	0.2 ns
**Cord blood pH**	Venous (N)	7.28±0.02 (30)	7.27±0.03 (6)	0.3 ns
	Arterial (N)	7.34±0.01 (30)	7.32±0.01 (6)	0.9 ns

For isolation of CPA, the amnion was gently peeled away by hand to reveal the network of arteries at the chorionic surface. An artery in isolation from a neighbouring vein was identified by following the point of cord insertion and tracking smaller branches of the selected artery. The fourth order branch of each CPA was used for the study and was dissected intact and free of adherent tissue. Three separate samples from each placenta were taken in this way. Following fine dissection to remove any connective tissue, CPA were transferred to physiological salt solution (PSS; composition in mmol/l: 119 NaCl, 4.7 KCl, 25 NaHCO_3_, 2.5 MgSO_4_, 1.6 CaCl_2_, 1.2 KH_2_PO_4_, 5.5 glucose, 0.034 EDTA pH 7.4) at room temperature, 21–26°C) and used immediately for wire myography.

### Wire myography

Once all connective tissue had been removed, CPA were transferred to cold (4–6°C PSS) and used immediately for wire myography. Each vessel was mounted onto the free end of a 50 µm tungsten wire before securing with a second fixing screw. A second wire was passed through the lumen and fixed to the opposite jaw so that the two wires were in parallel and any remaining connective tissue removed. The remaining vessel segments were mounted in the same way and left to equilibrate and heat to 37°C before normalisation. The effective pressure was determined for each vessel segment and normalized to an effective pressure of 5.1 kPa and set at a circumference of 0.9. The resting pressure of 5.1 kPa was applied to closely match the transluminal pressure recorded across the intervillus space of the placenta ([22], [23]. CPA were preconstricted with the thromboxane mimetic U46619 (1 nM–1 µM in PSS), and responses to a number of ion channel and vasoactive modulators investigated. The tissue baths were continuously perfused with 5% O_2_, 5% CO_2_, 90% N_2_ (BOC, UK) and maintained at 37°C. The effects of extracellular acidification were assessed by applying decreasing doses of 1M lactic acid in 0.2 units (pH 7.6–6.4) to the bath, in the presence or absence of various ion channel modulators and vasoactive agents. The pH of the bath solutions was routinely checked. Each compound was added directly to the chamber twenty minutes prior to stimulation with U46619. The baseline tension of each vessel was assessed pre- and 5 mins post-addition of each pharmacological agent. One vessel in each experiments to which no drugs were added was used as a time control or as a vehicle control where the solvent alone was added to differentiate specific drug-induced effects. Concentrations of agents used were based on experiments performed in our lab along with published literature.

### Placental VSM primary culture

VSM were cultured using explants of CPA based on the methodology of Leik *et al.*, [24]. One mm^2^ square pieces of artery were excised and each piece placed in the well of a 12-well plate with the endothelium facing downwards on to the plastic surface. Dulbecco's Modified Eagle Medium (DMEM; containing 10% (v/v) heat-inactivated fetal bovine serum (FBS), 100 units/ml penicillin,100 µg/ml streptomycin) were added to each well and plates maintained in a humidified incubator in a 5% CO_2_/air environment. Apart from regular media changes, culture plates were left for a period of 3–4 weeks at which point outgrowths could be subcultured by simply removing the explant and trypsinising (0.025% in calcium - and magnesium -free Hanks Balanced Salt Solution, HBSS) the sample followed by washing in serum-containing DMEM to inactivate trypsin. The cell pellet was washed again and centrifuged at 200 g before resuspending cells for transfer into a T-25 tissue culture flask. Myometrial biopsies from non-laboring patients were enzymatically dispersed immediately after collection [25] to produce single, smooth muscle cells and cultured to be used as a positive control for immunofluorescence studies.

### Confocal immunofluorescence

Placental and myometrial smooth muscle cells were cultured on glass coverslips in DMEM (with 10% heat-inactivated fetal bovine serum and penicillin/streptomycin) until 70% confluent. Culture media was aspirated then cells fixed with 4% w/v paraformaldehyde (pH 7.4) for 5 mins at 4°C. Fixative was then gently removed and 3% bovine serum albumin (BSA)/1% glycine in PBS added to cells for 15 mins followed by blocking with a mixture of 10% goat and 10% chick serum in PBS. After 1 hour, the blocking solution was aspirated and cells incubated with 200 µl of primary antibody against the channels TASK-3 (1∶50), TREK-1 (1∶100) or TWIK-2 (1∶50), and CaV1.2 (1∶50) and left overnight at 4°C with gentle shaking. A negative control was also used where primary antibody was replaced with either control IgG or antibody was incubated with excess control peptide according to the manufacturer's instructions before adding to the cells. Following removal of the primary antibody, the coverslips were coated with either anti-mouse FITC or anti-rabbit TRITC conjugated secondary antibodies (1∶5000) in 10% goat serum in PBS for 1.5h in the dark with gentle shaking then mounted in PBS∶glycerol. Mounted slides were placed face down on the slide stage and viewed using a Leica TCS SP2 confocal microscope with Acousto-Optical Beam Splitter (AOBS) scan head and a 63x glycerol immersion objective lens. Images were captured using three laser lines in single scans to eliminate any overlap between the channels. DAPI IF was viewed using an Ultraviolet laser (372 nm excitation and 385nm emission). FITC was viewed using 488 nm excitation with 505–530 emission (Argon laser) and TRITC was viewed with 546 nm excitation and 560 nm emission (Helium//Neon laser). Each individual sample was run at least twice for immunofluorescence studies.

### Drugs and solutions

All reagents were obtained from Sigma Aldrich (UK). Drugs used were curcumin, L-methionine and ruthenium red as TREK-1 inhibitors; bupivacaine, methanadamide, copper chloride, lidocaine and zinc chloride to inhibit either TASK-1 or TASK-3; quinidine and barium chloride as inhibitors of TWIK channels, iberiotoxin to block BK_Ca_ channels; apamin as an SK_Ca_ channel blocker and nifedipine for L-type Ca^2+^ channels. Concentrations used were based on published data or preliminary experiments carried out in our lab. Drugs were prepared daily from fresh stock solutions or from frozen stocks. Curcumin and ruthenium red were prepared in ethanol while nifedipine, quinidine and riluzole were added to DMSO then diluted in PSS. All other compounds used were soluble in PSS with pH adjusted to 7.4 prior to addition to the bath. For wire myography, the total addition of each compound to the bath was maintained at <1% of the total chamber volume. Time controls or vehicle controls (diluent only) were also included in each experiment. Ion channel antibodies were from Caltag Medsystems, UK.

### Data and statistical analysis

Concentration response curves were analysed by fitting the data to a 3-parameter logistic equation and compared by global curve fitting (GraphPad Software Inc Version 5, San Diego, CA, USA). The top and bottom plateaus were constrained and shared; the logEC_50_ values were compared with the control group. Normalcy was assessed using the Shapiro-Wilks normality test. Differences amongst responses were assessed using a one-way ANOVA with Bonferroni post hoc comparison across each concentration range used. Data are presented as mean ± SEM of n vessels used. A p value of <0.05 was considered to be statistically significant.

## Results

**Figure 1:**
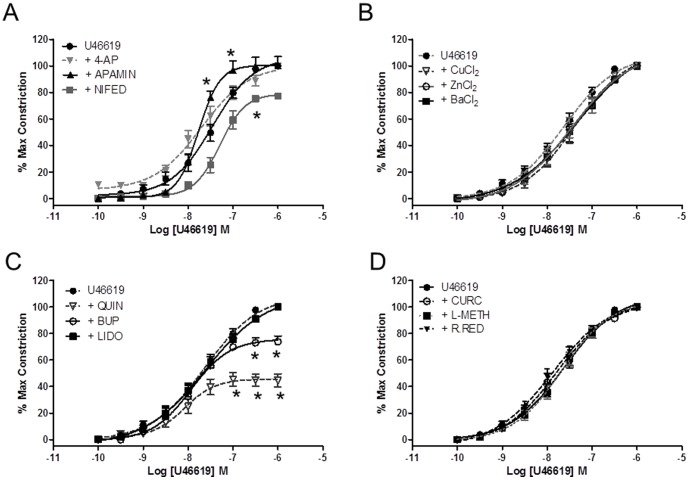
Modulation of the U4661-induced vasoconstriction by ion channel modulators. Wire myography of CPA was carried out in physiological salt solution with vessels mounted under tension. The effects of channel modulators were evaluated by preincubating CPA with test drug and comparing responses of CPA preconstricted with U46619. (A) The effect of voltage-gated channel blockers. Nifedipine caused a rightward shift in the dose-response curve. The Kv inhibitor 4-AP increased basal tension while apamin significantly altered the response to U46619 at higher concentrations. (B) The inorganic compounds BaCl_2_, CuCl_2_ and ZnCl_2_ had no effect on the U46619 curve (C) Bupivacaine and quinidine both reduced maximal vasoconstriction in CPA. No effect of non-specific TASK or TREK inhibitors was evident. For all graphs in this and subsequent figures, data are presented as mean ± SEM with p<0.05 (*) taken as a significant finding.

The effects of voltage-gated, calcium-sensitive and K2P channel modulators were investigated by direct application to resistance-sized CPA (vessel diameter 418±12 µm; n = 96) preconstricted with U46619. No significant effect (p>0.05) of 10 µM glibenclamide, a K_ATP_ channel inhibitor (n = 9) on the U46619 concentration-response curve was observed. Pre-incubation with 10 µM apamin or 1 mM 4-AP enhanced basal tone and produced a leftward shift of the concentration-response curve (Fig. 1A). This shift was accompanied by altered log EC_50_ values of -7.8±0.1 for both apamin (n = 9) and 4-AP (n = 9) compared with −7.5±0.1 for U46619 alone (n = 12). The CaV1.2 blocker nifedipine displaced the control curve to the right with an EC_50_ of −7.3±0.1 (n = 9) when vessels were challenged with U46619 concentrations above 10^−8^M. Nifedipine (10 µM) also reduced the maximal response to 79.01% (n = 9). The inorganic compounds CuCl_2_, ZnCl_2_ and BaCl_2_ (n = 9 for all), each at a bath concentration of 1 mM, had no effect on CPA responses to U46619-induced vasoconstriction (Fig. 1B). The lack of effect of the local anesthetic lidocaine (100 µM), was in contrast to a decrease in maximal contraction by 26.1±4.2% and 55.4±3.7% respectively elicited by pre-incubation with either 200 µM bupivacaine (n = 9) or 200 µM quinidine (n = 9; Fig. 1C). Neither L-methionine (n = 9), ruthenium red (n = 9) nor curcumin (n = 9) significantly altered the contractile performance of U46619-induced vasoconstriction (p>0.05; Fig. 1D).

**Figure 2:**
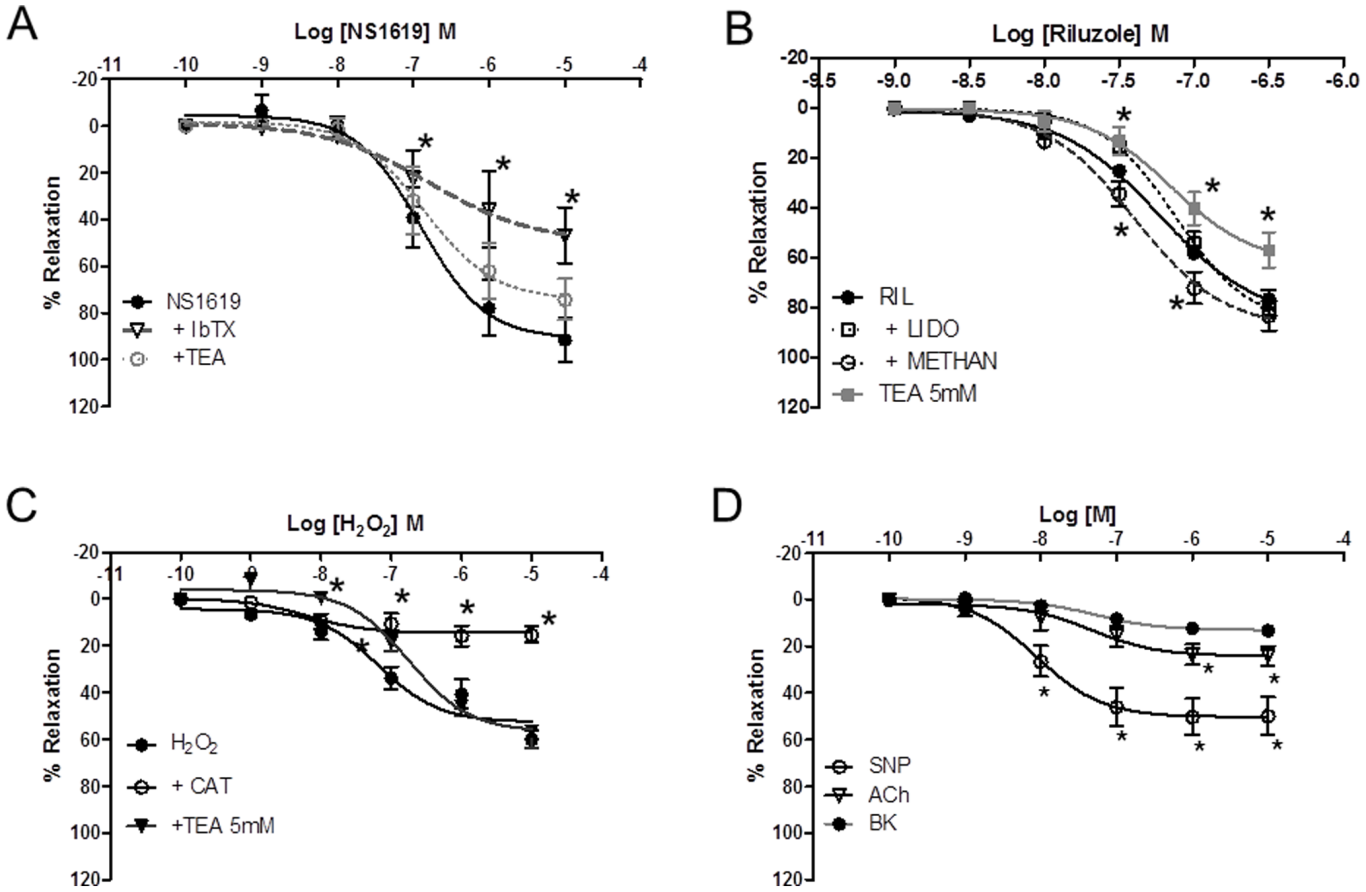
CPA relaxant effects induced by vasomodulators. (A) The BK_Ca_ channel opener NS1619 produced relaxation of preconstricted CPA. The response was significantly inhibited by iberiotoxin but not by TEA. (b) Riluzole also caused relaxation that was unaffected by lidocaine, and methanandamide but significantly inhibited by TEA (C) Catalase inhibited the H_2_O_2_ vasorelaxation while TEA had no effect on the maximal relaxation but caused a rightward shift of the curve (D) SNP, bradykinin and ACh induced vasorelaxation to differing extents.

On examining vasodilatatory responses of U46619-preconstricted vessels, the BK_Ca_ channel opener NS1619 (10-^10^ - 10^−5 ^M) resulted in a significanµt, concentration-dependent relaxation of 91.5±8.3% (n = 4), an effect that was maximally inhibited to 74.2±8.4% with TEA (n = 4; p>0.05) and even further (46.9±11.9%; n = 4) following pretreatment with the BK_Ca_ channel antagonist iberiotoxin. These responses differed significantly from controls (p<0.05; Fig. 2A). The widely-used TREK-1/TRAAK channel opener, riluzole, also caused effective relaxation (83.0±3.8%) of preconstricted arteries (n = 18), which was inhibited by 5 mM TEA (56.8±7.1%; n = 6) but unaffected by 100 µM methanandamide (n = 6), 100 µM lidocaine (n = 6; Fig. 2B), 10 µM ruthenium red (n = 6), L-methionine (n = 6), 10 µM curcumin (n = 13), 200 µM bupivacaine (n = 6), 200 nM IbTX (n = 4), 1 mM 4-AP (n = 6) and 10 µM apamin (n = 6; data not shown).

Since H_2_O_2_ may also relax arteries, we tested the effects of catalase on the H_2_0_2_-mediated relaxation of U46619-preconstricted CPAs. Vasorelaxation to H_2_O_2_ was considerably attenuated by catalase at 10-^7 ^M and 10^−5 ^M (n = 5; Fig. 2C). TEA with a log IC_50_ of −3.8±0.1% demonstrated an inhibition of the H_2_O_2_ response at the lower doses of 10^−8^–10^−7 ^M (p<0.05) and also at 10^−5^M (p<0.05; n = 4). In contrast, SNP caused 49.9±7.9% relaxation of the U46619 response which was greater than that induced either by ACh (24.2±4.2%) or bradykinin (13.2±2.1%) as shown in Fig. 2D. Direct application of H_2_O_2_ produced variable, transient and desensitising responses when added directly to CPA but these were not investigated further. The non-specific TREK-1 blocker curcumin [26], also known for its antioxidant properties, produced no significant shift in the response of CPA to H_2_O_2_ (n = 5) while iberiotoxin had little effect on the vasodilatation evoked by application of H_2_O_2_.

### The effect of acidification

**Figure 3:**
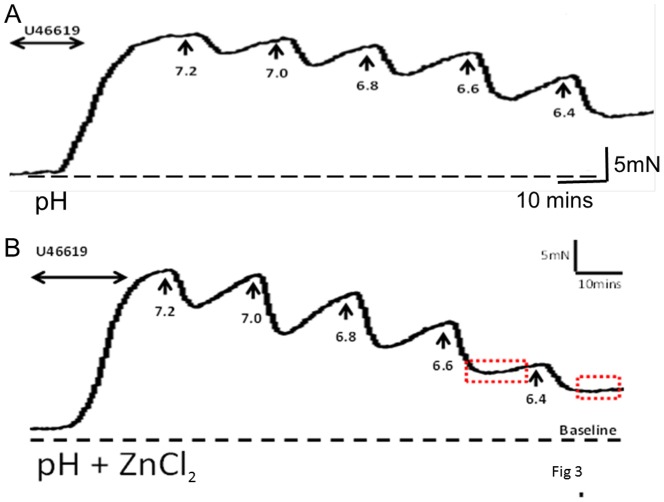
The effects of pH on CPA of normal vessels. Incremental decreases in pH were introduced by direct addition of lactic acid to preconstricted vessels. (A) Transient relaxations were immediately induced followed by recovery and a return of tone. (B) Preincubation with ZnCl_2_ enhanced the relaxant effects of pH as well as inhibiting the recovery phase shown by the boxed regions.

**Figure 4:**
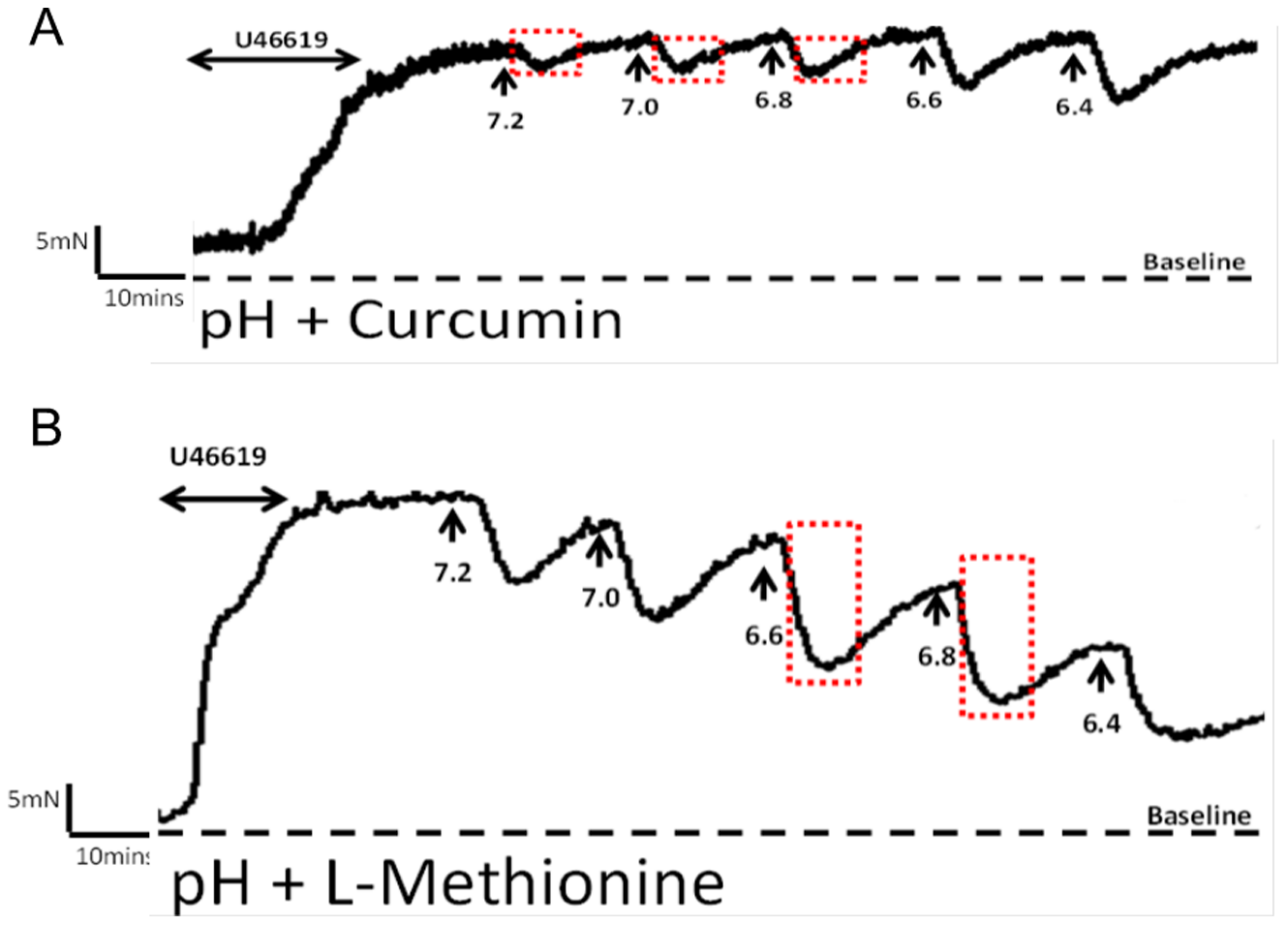
The effects of TREK-1 modulators. (A) 10 µM Curcumin inhibited both the maximal relaxant effect by pH and accelerated the recovery phase. (B) pH effects were potentiated by 1 mM L-methionine shown by the boxed area.

**Figure 5:**
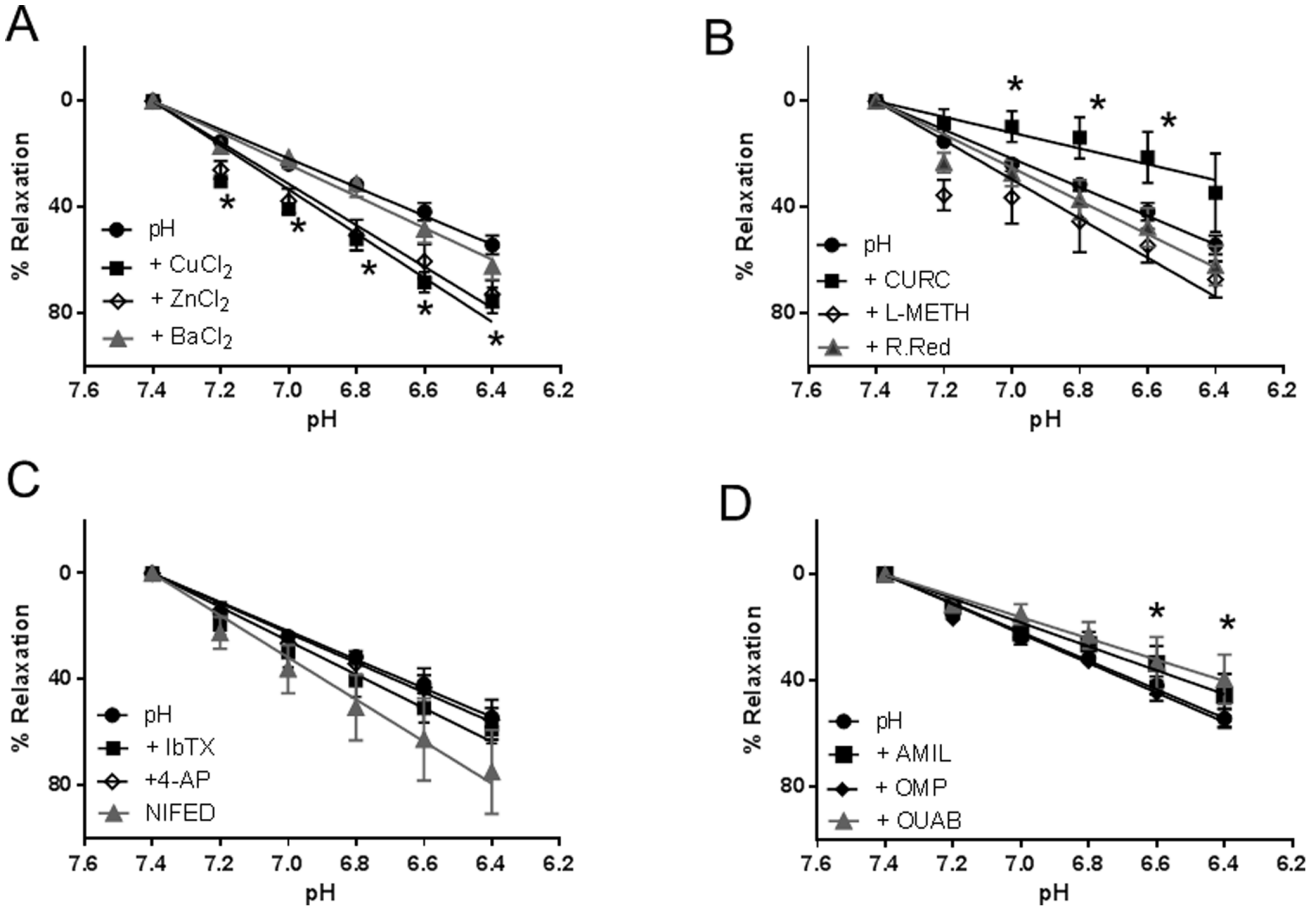
The effect of channel modulators on the pH response of U46619-preconstricted vessels. (A) 1 mM BaCl_2_ had no effect on relaxation while both 1 mM CuCl_2_ and 1 mM ZnCl_2_ significantly increased the relaxation evoked. Curcumin inhibited relaxation while 10 µM ruthenium red had no effect (C) L-methionine induced relaxation at (C) 10 µM Nifedipine inhibited the response while 1 mM 4-AP and 200 nM IbTX had no effect, (D) Neither 10 µM amiloride, 10 µM omeprazole nor 50 µM ouabain had any effect on the pH response while ouabain was seen to relax CPA

Incremental decreases in extracellular pH (7.4–6.4) by addition of lactic acid resulted in concentration-dependent relaxation of U46619-preconstricted CPA (Fig. 3, top trace). The vascular responses were transient nature, characterised by an immediate downward deflection followed by a return to baseline that we have termed the recovery period, as seen in Fig. 3 (top trace). Maximal relaxation (54.2±3.5%) of the U46619-preconstriction was observed at a pH of 6.4 (n = 16). In order to investigate the contribution of channel families underlying the relaxation induced by pH, a variety of channel blockers were tested. Pre-incubation of CPA with 1mM ZnCl_2_ produced a greater degree of relaxation (75.1±4.7%; n = 6) than pH alone and prolonged the recovery period as pH was lowered (Fig. 3B), achieving significance at pH<7.0 (Fig. 5A). Curcumin inhibited the relaxant effect induced by pH (Fig. 4A; Fig. 5B) while pre-incubation with L-methionine, an inhibitor of stretch-activated channels thought to be TREK-1, accelerated the downward deflection of the relaxant response and increased the magnitude of the response as pH was reduced (Fig. 4B; Fig. 5B). CuCl_2_ also affected the recovery phase inhibiting the contractile effect and enhancing relaxation to 72.8±4.9% (n = 6; Fig. 5A). BaCl_2_ had no significant effect (p>0.05) on pH responses (Fig. 5A). Neither iberiotoxin nor 4-AP had any significant effect on the pH response while nifedipine significantly increased the relaxation to pH to 74.0±5.0% (p<0.05; Fig. 5C). Ruthenium red (Fig. 5B), amiloride and omeprazole had no effect on the response of CPA to pH although a slight effect of ouabain was significant (p<0.05) at lower pH values compared with responses to pH alone (Fig. 5D).

**Figure 6:**
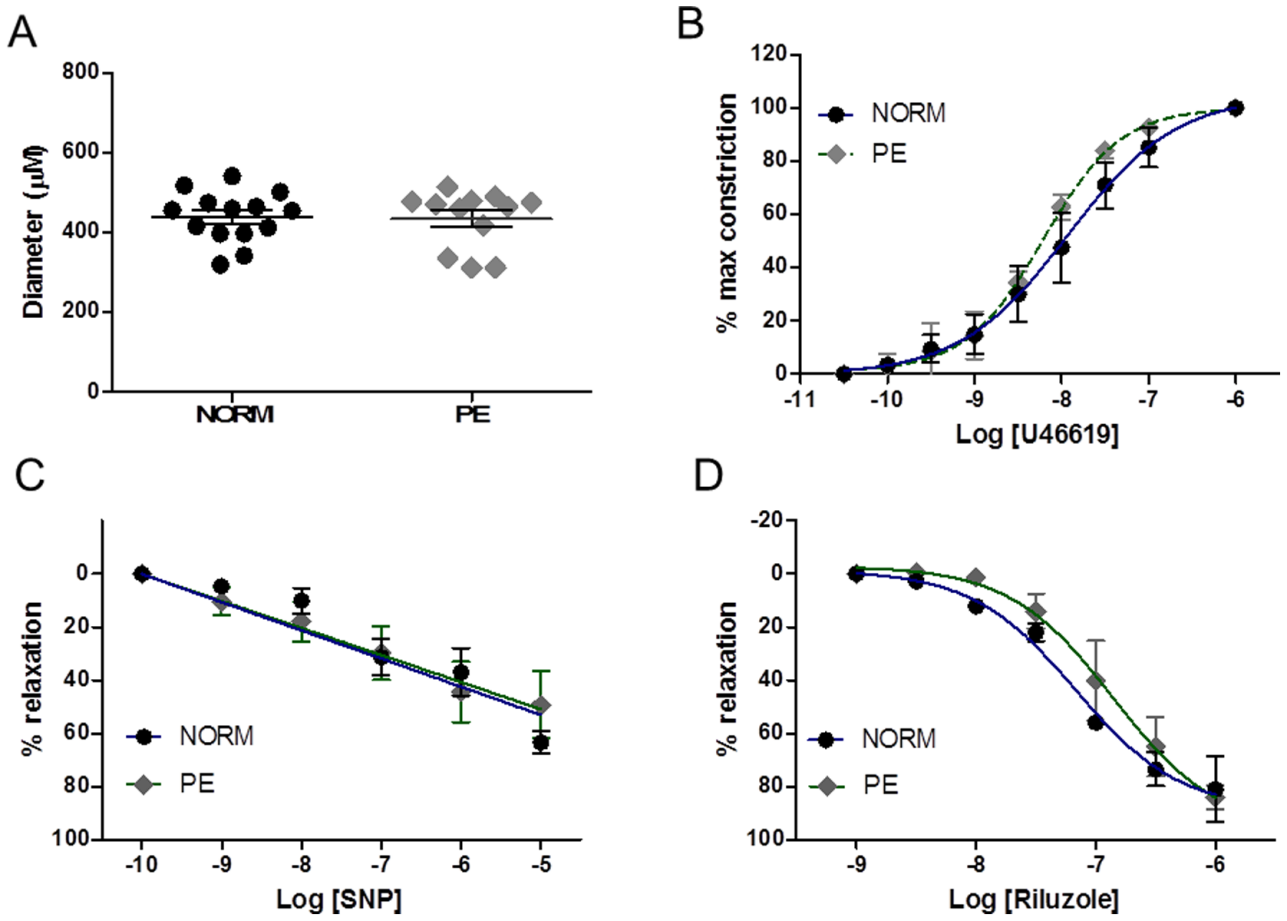
Comparison of *ex vivo* responses of CPA vessels from pre-eclamptic patients with those of normal pregnancy. (A) CPA isolated from pre-eclamptic (PE) and normal (NORM) pregnancies used for myography had similar diameters (p>0.05). (B) Concentration-response curves of CPA of PE and NORM patients exhibited similar responses to U46619. Vasorelaxation of vessels to SNP (C) and riluzole (D) was unchanged between normal and PE pregnancies (p>0.05).

**Figure 7:**
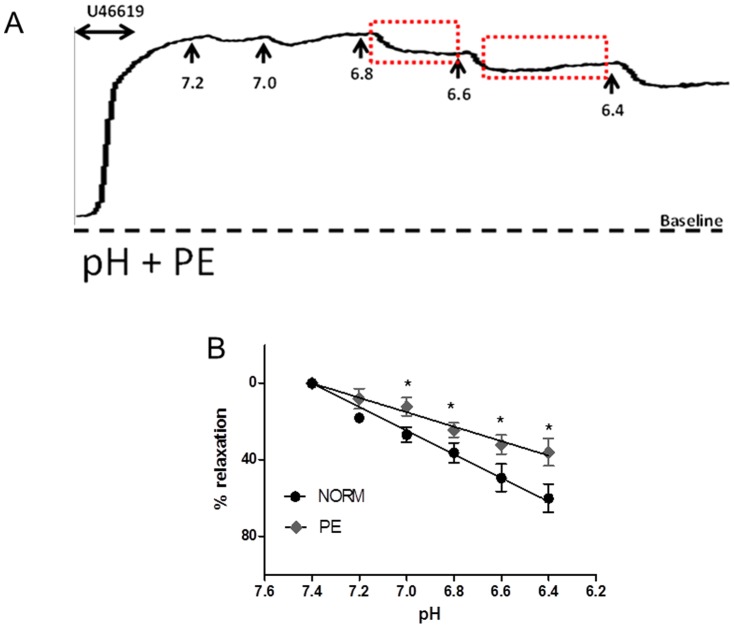
Effects of pH on vessels from women with pre-eclampsia. (A) Representative trace of CPA from a pre-eclamptic patient to stepwise addition of pH. Lower pH reduced the ability of the vessel to return to baseline (red box). (B) Concentration-response curve to pH showed a significant inhibition in the relaxant response of PE samples across the lower pH range.

CPAs from patients with pre-eclampsia obtained at ELLSCS were also included in some of our functional studies. Fig. 6A shows that using the same 4th branch of the CPA from PE placental samples, vessel diameter was not found to be significantly different to CPAs for normotensive patients (p>0.05). Neither was the dose-response curve to U46619 of CPA significantly different between normal and PE vessels (p>0.05; Fig. 6B). Vascular responses, in terms of maximal relaxation and log EC_50_ values to the relaxants sodium nitroprusside log EC_50_s (−5.5±0.1 and −5.7±0.1 for normal and PE respectively; Fig. 6C) and riluzole (log EC_50_s −6.6±0.1 and −6.7±0.1; for normal and PE respectively; Fig. 6D) were also unaltered between the two groups (p>0.05 in both cases). Interestingly PE vessels demonstrated an attenuated vasorelaxant response on acidification compared with normal vessels (Fig. 7A and Fig. 7B).

**Figure 8:**
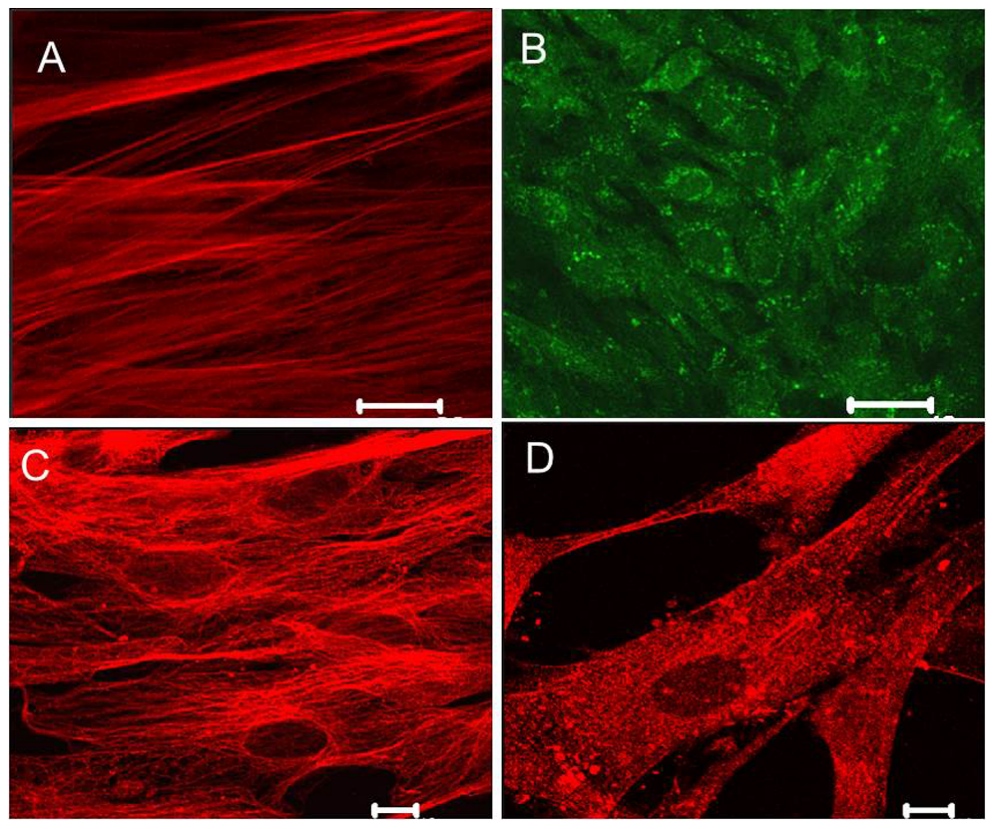
Immunolocalisation of placental ion channels. Cultured CPA were fixed then incubated with primary antibody to the channel of interest then visualised. Immunofluorescence for (A) TREK-1; scale bar 10 µm, (B) TASK-3; scale bar 40 µm (C) CaV1.2 channels; scale bar 10 m, was apparent. Patterns of expression varied with TREK-1 characterised by linear expression while TASK-3 was perinuclear. (D) Representative TWIK-2 expression in CPA; scale bar 10 µm.

Protein expression and localisation of TREK-1 (n = 12; Fig. 8A) and TASK-3 (Fig. 8B) was confirmed by confocal immunofluorescence compared with control samples where primary antibody was replaced with the relevant control IgG. TREK-1 immunofluorescence was characteristically linear across the cell membrane (Fig. 8A). Representative images show TASK-3 (Fig. 8B) expression was abundant around perinuclear regions but was also membrane-associated (Fig. 8B). CaV1.2 (corresponding to LTCC) immunofluorescence was clearly observed along VSM of CPA (n = 10; Fig. 8C) and intenseTWIK-2 expression was also noted (n = 5; Fig. 8D).

## Discussion

Excess protons are potentially harmful to cells as they can cross the cell membrane with ease and interfere with key cellular functions and pathways. This has important consequences in the placenta which needs to maintain continuous blood flow through which effective fetomaternal transfer for elimination of waste metabolites into the maternal circulation is achieved. Acidic pH may also lead to chronic metabolic inhibition as ATP levels fall adding to the pH insult [27]. Levels of metabolic acids such as lactic acid have been shown to increase when tissue perfusion is low as may occur during certain pregnancy disorders or during uterine contractions accompanying normal labour [28], [29]. Umbilical cord arterial pH values in normal term infants lie in the range 7.2–7.3 while cord arterial pH values below a threshold of 7.0 are associated with perinatal mortality and morbidity as well as cerebral palsy [30]. We used lactic acid for the experiments described herein to demonstrate that lowering extracellular pH presents a significant H^+^ challenge resulting in an immediate and initial loss of arterial tone in placental resistance vessels. This response is transient and reversible as the vessel recovers its contractile function probably as a consequence of buffering agents within the saline. These effects of pH afforded an opportunity to investigate the specific ion channels expressed in CPA as well as the main subtypes underlying the pH responses.

In most vascular preparations, lowering pH results in vasodilatation and is consistent with the responses we observed in of CPA. Our findings corroborate observations by others on the role of LTCC in the placenta with the dihydropyridine blocker, nifedipine, inhibiting the U46619 constriction [6] in accordance with its role in HFPV ([7] [31]. Pre-treatment with nifedipine enhanced the response to acidification and may be linked to the reduced contractile effect of U46619 produced in the presence of nifedipine. CPA were also able to produce a transient response upon stimulation with lactic acid in the presence of this LTCC channel blocker indicating other channels may underlie this response.

Vascular effects implicating the BK_Ca_ channel in CPA function are interesting. Although we failed to demonstrate a role for this channel either on basal tone or pH responses, the BK_Ca_ channel opener NS1619 evoked a potent iberiotoxin-sensitive relaxation in CPA compared to that seen with SNP suggesting it may form part of an important feedback loop that counteracts depolarisation. A lack of effect of low pH on BK_Ca_ while consistent with data from other vascular beds [32], [33] arteries contrasts with findings in cerebral smooth muscle such as parenchymal brain arteries where acidosis is linked with vasodilation through BK_Ca_ channels [34], [35] therefore this channel does not appear to play [6] a direct role in combatting acidosis or ischemia. Our immunofluorescence observations confirmed the localisation of both BK_Ca_ and LTCC proteins to the membrane of CPA supporting their functional contributions to the control of placental arterial tone [6], [7], [15].. However, as our experiments relied on a month-long culture period of explants before sufficient single VSM cells were obtained for immunofluorescence, expression may not reflect channel expression in native cells, largely due to channel expression being modified by *in vitro* conditions including oxygen tension.

The effects of apamin on CPA are consistent with a role for SK_Ca_ channels reported to be present in myocytes of CPA [14]. However the concentration used in our study was greater than required for specific blockade of SK_Ca_ and therefore our observation is of limited value. The fact that pH did not alter the effect of the U46619-induced vasoconstriction observed in the presence of apamin suggests no role for these channels in acidosis. The modified CPA response in the presence of 4-AP lends further support for the role of Kv channels in regulation of placental tone [7] but unaffected by pH.

We provide evidence for the first time for K2P channel protein expression in CPA of TREK-1, TWIK-2 and, TASK-1. TREK-1 currents were initially reported in rat brain slices where this channel has the highest expression levels [36]. Subsequent studies have shown that TREK-1 is also widely expressed in many tissue types [37], [38]. Its capacity to control cellular function through its sensitivity to diverse stimuli including temperature, intracellular pH, lipids and stretch [39]–[41] identifies it as a target for the convergence of multiple signals and therefore is of potential therapeutic interest. TREK-1 is also present in cerebral arteries where it is a target for the action of polyunsaturated fatty acids and underpins the neuroprotection attributed to this group of lipids [42], [43]. The importance of the TREK-1 response to stretch was shown in a recent using bladder smooth muscle cells [44]. The bladder wall stretches during the process of filling with stretch-sensitive ion channels implicated in maintaining quiescence to prevent unregulated contraction of the bladder wall. In bladder myocytes, L-methionine, but not its inactive enantiomer D-methionine, blocked the response by bladder myocytes to stretch with TREK-1 the most likely stretch-dependent channel mediating mechanotransduction [44]. In our study, L-methionine potentiated instead of blocking vasorelaxation indicating other routes for vasoactive effects of this amino acid. One of these may be through the metabolism of L-methionine to L-cysteine which replenishes sulfhydryl pools necessary for biotransformation of glyceryl trinitrate [45].

Other non-specific modulators of stretch-dependent K^+^ channels had varying effects on the pH response of CPA. Curcumin, the active ingredient of turmeric has been shown to inhibit bovine TREK-1 channels, coupling this response to the depolarisation required for cortisol secretion from adrenocortical cells [46]. This observed inhibition induced by low pH is arguably consistent with blockade of a K^+^ conductance in CPA. Curcumin's effects include its antioxidant properties as well as inhibition of the transcription factor nuclear factor κ-B which given the immediacy of the response to low pH is unlikely to be the mechanism underlying the effects of this phytochemical on transcription. Interestingly, the neuroprotectant riluzole which inhibits voltage-gated sodium channels and SK_Ca_ channels [47] has dual effects on TREK-1 channels where it serves to activate and inhibit TREK-1 currents [48]. Its action on CPA was to produce relaxation of preconstricted arteries that could occur via SK_Ca_ channel or TREK-1 modulation. In contrast to our findings where the riluzole effect was not altered by pH, the response of mouse taste-bud cells in to citric acid, was potentiated by riluzole via TREK-1 blockade by acid-induced cell depolarisation [20]. The mechanosensitivity of TREK-1 may be closely linked with α-actin expression. Laurtizen [49]showed that TREK-1 activity could be altered following disruption of the α actin network in embryonic striatal neurons with implications for synaptogenesis. The pattern of TREK-1 staining noted in cardiac ventricular myocytes is thought to detect longitudinal stretch [50]. Given this evidence, the colocalisation of TREK-1 and α-actin that we observed suggests a role for this channel in mechanotransduction in response to vessel stretch or vasoconstriction.

Our observation that both CuCl_2_ and ZnCl_2_ enhanced the relaxant effect of CPA at low pH does not point to the effect of acidification being mediated by K2P channels since blocking them would be expected to result in reduced relaxation. In fact, the increase in vessel tone seen during the recovery period immediately after acidification was blunted by both CuCl_2_ and to a greater extent with ZnCl_2_. The effects of these inorganic ions are difficult to tease apart due to their varied effects at several ion channels. Electrophysiological recordings of channels that exhibit both pH and zinc sensitivity have shown that application of 100-200µm of ZnCl_2_ can reduce TASK currents by 50% [51]. At these concentrations, we saw no change in the baseline response of CPA to ZnCl_2_ and opted to use the higher dose of 1mM in order to unmask any effect on isolated arteries. The response to ZnCl_2_ can also be considered by the effects the trace element has on Ca^2+^ channel currents. ZnCl_2_ inhibits the entry of Ca^2+^ into cells and this may explain why we observed a blunting of the contraction that followed the initial relaxation. Ischemia is an important modulator of Zn^2+^ levels and an increase in free unbound Zn^2+^ (where it is normally bound to protein) can alter neuronal excitability [52]. Zn^2+^ can inhibit Ca^2+^ and Na^+^ channel currents (along with TASK-1) to alter neurotransmitter release and in this context, the failure of the vessels to recover from relaxation in the presence of ZnCl_2_ suggests a potential blocking effect on Ca^2+^ channels involved in depolarisation.

Copper ions can bind with high affinity to cysteine, histidine, or glutamate residues which can lead to a dissociation of disulfide bridges which in K2P channels hold the pore-forming domains in place. Gruss [51] examined the role of both ions in modulating HEK-293 cells stably transfected with human TREK-1 and TASK-3. It was shown that copper activated TREK-1 while an inhibition was seen with TASK-3 [51]. With respect to pH, TASK channels open at the resting membrane potential and are a target for anaesthetics and extracellular pH. TASK-1 mRNA has been shown to be high within the placenta ([17], [53]. External acidification will inhibit both TASK 1 and TASK-3 which would have an opposite effect to the relaxation we observed in CPA but studies have shown that the exact pH value has contrasting effects on each ion channel subtype. TASK-1 is inhibited at pH 7.1–7.4 while TASK-3 is inactive at pH <6.7 [17]. The two ion channels in CPA also appeared to colocalise and given their range of pH sensitivities suggests that TASK-3 and TASK-1 may be differentially activated. Methanandamide has also been shown to inhibit both TASK-1 and TASK-3 channels but in our hands a meagre effect of methanandamide on the riluzole-mediated vasorelaxant effect, unaffected by pH, was observed. Since methanadamide is an agonist at endocannabinoid receptors CB1 and CB2 [26], [54], the relevance of our observation is limited.

The broad-acting K2P channel antagonist quinidine has as its most likely targets the K2P channels TWIK-2, TREK-1, TREK-2 and TASK-2. The tissue distribution of human TWIK-2 is high in the placenta along with the aorta, oesophagus, stomach and spleen [19]. Expression for the ion channel was detected in CPA and mRNA for the K2P members TWIK 1/2 was present in mouse taste-buds with TWIK 1/2 levels evidently the highest [20]. The quinidine block of CPA in the absence of pH with no change on acidification indicates that TWIK-2 channels are not implicated in responses to pH but supports a role for the channel in placental vascular function.

Other likely candidates implicated in the pH response of CPA are the acid-sensing family of ion channels (ASIC). H^+^ ions are removed from the cell via ATP dependent Na^+^ transporters [55]. A small effect of ouabain (Na^+^/K^+^ ATPase inhibitor), and absence of an effect of the NHE inhibitor omeprazole along and the epithelial Na^+^ channel blocker amiloride suggest no role for ASICs in CPA tone when the extracellular face was acidified. However, an attenuation of the acidic pH response when NaCl was replaced with choline chloride suggests Na^+^ are potentially involved in the recovery from acidic pH insults and could involve non-selective cations channels including those of the transient receptor potential family (TRPs) that are pH-sensitive [56].

The functional responses from a small population of CPA from PE placenta were examined as part of the study since myometrial arteries from pre-eclamptic women display altered endothelium-mediated vasorelaxation [57]. The main difference between vessels from PE and normal pregnancies was seen on exposure to acidic pH with PE samples exhibiting a reduced loss in tone across the same dose range. This diminished ability to relax in response to an acidic pH stimulus could potentially be harmful as the excess H^+^ cannot be efficiently buffered in placental blood. This would carry the risk of spreading the low pH insult to other areas of the placenta and more importantly expose the fetus to the same stress. It was significant that the pH response was blunted in PE samples and suggests the vessels may be less sensitive to low pH or may reflect the altered vasoconstriction seen in PE placentae. In fact, it has been shown that severe PE may predispose the fetus to acute hypoxia and fetal acidosis resulting in fetal distress [58]. Reactive oxygen species generated as a result of hypoxia (which often precedes acidosis) but also raised in pre-eclampsia [59], [60] also affect vascular function as shown by H_2_O_2_-mediated vasorelaxation of preconstricted CPA. This conflicts with the observations of no effect of H_2_O_2_ on CPAs preconstricted with U46619 [61]. Our limited data set using CPAs of pre-eclamptic women provides an insight into the altered vascular function of adverse pregnancy samples but this study needs to be expanded to better understand the importance of these differences.

This study reports for the first time the expression of key members of the K2P channel family, TWIK-2, TASK-3 and TREK-1 in intact CPA and within cultured smooth muscle cells isolated from CPA. We also report that low pH causes vasorelaxation of CPA. In an *in vivo* context, extracellular acidification would improve perfusion possibly to minimise local acidic insults. Our work and that of others working with K2P channels is hampered by the lack of specific K2P channel antagonists. As a result, we may have been observing the effects of blocking more than one ion channel compensated by activation of another to maintain resting membrane potential. Responses to acidic pH will activate a number of proton-sensing ion channels however the data provided imply that no single ion channel is the target of extracellular pH. Importantly, functional responses may also reflect the surgery beforehand. Allen showed that anaesthetics used during ELLSCS can alter the function of placental vessels [62]. It is well known that ion channels especially some K2P channels are modulated directly by anaesthetics [37], [43], [63] and therefore samples should also be studied following vaginal delivery where alternative forms of pain relief are administered. Evidence is provided to suggest that compensatory mechanisms may exist in the placental vasculature to facilitate the response to acidic pH stress. Deciphering the potential role of K2P channels in CPA is complex and further work will need to make use of molecular methods such as RNAi to knockdown particular channels due to a lack of selective pharmacological agents active at subtypes of this channel family.

## References

[1] Omo-Aghoja L (2014) Maternal and fetal Acid-base chemistry: a major determinant of perinatal outcome. Ann Med Health Sci Res 4:8–17. 24669324 10.4103/2141-9248.126602 PMC3952302

[2] Bobrow CS, Soothill PW (1999) Causes and consequences of fetal acidosis. Arch Dis Child Fetal Neonatal Ed 80:F246–249. 10212094 10.1136/fn.80.3.f246 PMC1720942

[3] Parratt J, Taggart M, Wray S (1994) Abolition of contractions in the myometrium by acidification in vitro. Lancet 344:717–718. 7915777 10.1016/s0140-6736(94)92209-8

[4] Myatt L (1992) Control of vascular resistance in the human placenta. Placenta 13:329–341. 1438081 10.1016/0143-4004(92)90057-z

[5] Poston L (1997) The control of blood flow to the placenta. Exp Physiol 82:377–387. 9129952 10.1113/expphysiol.1997.sp004033

[6] Jakoubek V, Bibova J, Hampl V (2006) Voltage-gated calcium channels mediate hypoxic vasoconstriction in the human placenta. Placenta 27:1030–1033. 16368136 10.1016/j.placenta.2005.10.006

[7] Hampl V, Bibova J, Stranak Z, Wu X, Michelakis ED, et al (2002) Hypoxic fetoplacental vasoconstriction in humans is mediated by potassium channel inhibition. Am J Physiol Heart Circ Physiol 283:H2440–2449. 12388256 10.1152/ajpheart.01033.2001

[8] Howard RB, Hosokawa T, Maguire MH (1987) Hypoxia-induced fetoplacental vasoconstriction in perfused human placental cotyledons. Am J Obstet Gynecol 157:1261–1266. 3688085 10.1016/s0002-9378(87)80307-1

[9] Hoeldtke NJ, Napolitano PG, Moore KH, Calhoun BC, Hume RF Jr (1997) Fetoplacental vascular tone during fetal circuit acidosis and acidosis with hypoxia in the ex vivo perfused human placental cotyledon. Am J Obstet Gynecol 177:1088–1092. 9396899 10.1016/s0002-9378(97)70020-6

[10] Khong TY, De Wolf F, Robertson WB, Brosens I (1986) Inadequate maternal vascular response to placentation in pregnancies complicated by pre-eclampsia and by small-for-gestational age infants. Br J Obstet Gynaecol 93:1049–1059. 3790464 10.1111/j.1471-0528.1986.tb07830.x

[11] Hu XQ, Zhang L (2012) Function and regulation of large conductance Ca(2+)-activated K+ channel in vascular smooth muscle cells. Drug Discov Today 17:974–987. 22521666 10.1016/j.drudis.2012.04.002 PMC3414640

[12] Ledoux J, Werner ME, Brayden JE, Nelson MT (2006) Calcium-activated potassium channels and the regulation of vascular tone. Physiology (Bethesda) 21:69–78. 16443824 10.1152/physiol.00040.2005

[13] Crane GJ, Gallagher N, Dora KA, Garland CJ (2003) Small- and intermediate-conductance calcium-activated K+ channels provide different facets of endothelium-dependent hyperpolarization in rat mesenteric artery. J Physiol 553:183–189. 14555724 10.1113/jphysiol.2003.051896 PMC2343487

[14] Brereton MF, Wareing M, Jones RL, Greenwood SL (2013) Characterisation of K+ channels in human fetoplacental vascular smooth muscle cells. PLoS ONE 8:e57451. 23437391 10.1371/journal.pone.0057451 PMC3578819

[15] Wareing M, Bai X, Seghier F, Turner CM, Greenwood SL, et al (2006) Expression and function of potassium channels in the human placental vasculature. Am J Physiol Regul Integr Comp Physiol 291:R437–446. 16914430 10.1152/ajpregu.00040.2006

[16] Ketchum KA, Joiner WJ, Sellers AJ, Kaczmarek LK, Goldstein SA (1995) A new family of outwardly rectifying potassium channel proteins with two pore domains in tandem. Nature 376:690–695. 7651518 10.1038/376690a0

[17] Duprat F, Lesage F, Fink M, Reyes R, Heurteaux C, et al (1997) TASK, a human background K+ channel to sense external pH variations near physiological pH. Embo J 16:5464–5471. 9312005 10.1093/emboj/16.17.5464 PMC1170177

[18] Lesage F, Lazdunski M (2000) Molecular and functional properties of two-pore-domain potassium channels. Am J Physiol Renal Physiol 279:F793–801. 11053038 10.1152/ajprenal.2000.279.5.F793

[19] Chavez RA, Gray AT, Zhao BB, Kindler CH, Mazurek MJ, et al (1999) TWIK-2, a new weak inward rectifying member of the tandem pore domain potassium channel family. J Biol Chem 274:7887–7892. 10075682 10.1074/jbc.274.12.7887

[20] Richter TA, Dvoryanchikov GA, Chaudhari N, Roper SD (2004) Acid-sensitive two-pore domain potassium (K2P) channels in mouse taste buds. J Neurophysiol 92:1928–1936. 15140906 10.1152/jn.00273.2004

[21] Brown MA, Lindheimer MD, de Swiet M, Van Assche A, Moutquin JM (2001) The classification and diagnosis of the hypertensive disorders of pregnancy: statement from the International Society for the Study of Hypertension in Pregnancy (ISSHP). Hypertens Pregnancy 20:: IX–XIV. 10.1081/PRG-100104165 12044323

[22] Kleiner-Assaf A, Jaffa AJ, Elad D (1999) Hemodynamic model for analysis of Doppler ultrasound indexes of umbilical blood flow. Am J Physiol 276:H2204–2214. 10362705 10.1152/ajpheart.1999.276.6.H2204

[23] Wareing M, Crocker IP, Warren AY, Taggart MJ, Baker PN (2002) Characterization of small arteries isolated from the human placental chorionic plate. Placenta 23:400–409. 12061856 10.1053/plac.2002.0825

[24] Leik CE, Willey A, Graham MF, Walsh SW (2004) Isolation and culture of arterial smooth muscle cells from human placenta. Hypertension 43:837–840. 14967841 10.1161/01.HYP.0000119191.33112.9c

[25] Chanrachakul B, Pipkin FB, Khan RN (2004) Contribution of coupling between human myometrial beta2-adrenoreceptor and the BK(Ca) channel to uterine quiescence. Am J Physiol Cell Physiol 287:C1747–1752. 15329337 10.1152/ajpcell.00236.2004

[26] Kuner P, Schubenel R, Hertel C (1998) Beta-amyloid binds to p57NTR and activates NFkappaB in human neuroblastoma cells. J Neurosci Res 54:798–804. 9856863 10.1002/(SICI)1097-4547(19981215)54:6<798::AID-JNR7>3.0.CO;2-T

[27] Larcombe-McDouall JB, Harrison N, Wray S (1998) The in vivo relationship between blood flow, contractions, pH and metabolites in the rat uterus. Pflugers Arch 435:810–817. 9518510 10.1007/s004240050588

[28] Wray S (1990) The effects of metabolic inhibition on uterine metabolism and intracellular pH in the rat. J Physiol 423:411–423. 2388156 10.1113/jphysiol.1990.sp018030 PMC1189765

[29] Quenby S, Pierce SJ, Brigham S, Wray S (2004) Dysfunctional labor and myometrial lactic acidosis. Obstet Gynecol 103:718–723. 15051564 10.1097/01.AOG.0000118306.82556.43

[30] Malin GL, Morris RK, Khan KS (2010) Strength of association between umbilical cord pH and perinatal and long term outcomes: systematic review and meta-analysis. BMJ 340:c1471. 20466789 10.1136/bmj.c1471 PMC2869402

[31] Jakoubek V, Bibova J, Herget J, Hampl V (2008) Chronic hypoxia increases fetoplacental vascular resistance and vasoconstrictor reactivity in the rat. Am J Physiol Heart Circ Physiol 294:H1638–1644. 18310520 10.1152/ajpheart.01120.2007

[32] Ishizaka H, Kuo L (1996) Acidosis-induced coronary arteriolar dilation is mediated by ATP-sensitive potassium channels in vascular smooth muscle. Circ Res 78:50–57. 8603505 10.1161/01.res.78.1.50

[33] Horiuchi T, Dietrich HH, Hongo K, Goto T, Dacey RG Jr (2002) Role of endothelial nitric oxide and smooth muscle potassium channels in cerebral arteriolar dilation in response to acidosis. Stroke 33:844–849. 11872913 10.1161/hs0302.104112

[34] Aalkjaer C, Peng HL (1997) pH and smooth muscle. Acta Physiol Scand 161:557–566. 9429665 10.1046/j.1365-201X.1997.00263.x

[35] Dabertrand F, Nelson MT, Brayden JE (2012) Acidosis dilates brain parenchymal arterioles by conversion of calcium waves to sparks to activate BK channels. Circ Res 110:285–294. 22095728 10.1161/CIRCRESAHA.111.258145 PMC3505882

[36] Meadows HJ, Benham CD, Cairns W, Gloger I, Jennings C, et al (2000) Cloning, localisation and functional expression of the human orthologue of the TREK-1 potassium channel. Pflugers Arch 439:714–722. 10784345 10.1007/s004249900235

[37] Terrenoire C, Lauritzen I, Lesage F, Romey G, Lazdunski M (2001) A TREK-1-like potassium channel in atrial cells inhibited by beta-adrenergic stimulation and activated by volatile anesthetics. Circ Res 89:336–342. 11509450 10.1161/hh1601.094979

[38] Magloire H, Lesage F, Couble ML, Lazdunski M, Bleicher F (2003) Expression and localization of TREK-1 K+ channels in human odontoblasts. J Dent Res 82:542–545. 12821716 10.1177/154405910308200711

[39] Maingret F, Patel AJ, Lesage F, Lazdunski M, Honore E (1999) Mechano- or acid stimulation, two interactive modes of activation of the TREK-1 potassium channel. J Biol Chem 274:26691–26696. 10480871 10.1074/jbc.274.38.26691

[40] Maingret F, Patel AJ, Lesage F, Lazdunski M, Honore E (2000) Lysophospholipids open the two-pore domain mechano-gated K(+) channels TREK-1 and TRAAK. J Biol Chem 275:10128–10133. 10744694 10.1074/jbc.275.14.10128

[41] Kang D, Choe C, Kim D (2005) Thermosensitivity of the two-pore domain K+ channels TREK-2 and TRAAK. J Physiol 564:103–116. 15677687 10.1113/jphysiol.2004.081059 PMC1456046

[42] Blondeau N, Petrault O, Manta S, Giordanengo V, Gounon P, et al (2007) Polyunsaturated fatty acids are cerebral vasodilators via the TREK-1 potassium channel. Circulation Research 101:176–184. 17556656 10.1161/CIRCRESAHA.107.154443

[43] Heurteaux C, Guy N, Laigle C, Blondeau N, Duprat F, et al (2004) TREK-1, a K+ channel involved in neuroprotection and general anesthesia. Embo Journal 23:2684–2695. 15175651 10.1038/sj.emboj.7600234 PMC449762

[44] Baker SA, Hennig GW, Han J, Britton FC, Smith TK, et al (2008) Methionine and its derivatives increase bladder excitability by inhibiting stretch-dependent K(+) channels. Br J Pharmacol 153:1259–1271. 18204472 10.1038/sj.bjp.0707690 PMC2275456

[45] Hussain AS, Abu-Zahra TN, Brien JF, Marks GS, Nakatsu K (1997) Thiol agents potentiate glyceryl trinitrate mediated relaxation of rabbit taenia coli: evidence for thiol-dependent biotransformation. Can J Physiol Pharmacol 75:807–811. 9315347

[46] Enyeart JA, Liu H, Enyeart JJ (2008) Curcumin inhibits bTREK-1 K+ channels and stimulates cortisol secretion from adrenocortical cells. Biochem Biophys Res Commun 370:623–628. 18406348 10.1016/j.bbrc.2008.04.001 PMC2394713

[47] Crane GJ, Garland CJ (2004) Thromboxane receptor stimulation associated with loss of SKCa activity and reduced EDHF responses in the rat isolated mesenteric artery. Br J Pharmacol 142:43–50. 15051624 10.1038/sj.bjp.0705756 PMC1574933

[48] Duprat F, Lesage F, Patel AJ, Fink M, Romey G, et al (2000) The neuroprotective agent riluzole activates the two P domain K(+) channels TREK-1 and TRAAK. Mol Pharmacol 57:906–912. 10779373

[49] Lauritzen I, Chemin J, Honore E, Jodar M, Guy N, et al (2005) Cross-talk between the mechano-gated K2P channel TREK-1 and the actin cytoskeleton. EMBO Rep 6:642–648. 15976821 10.1038/sj.embor.7400449 PMC1369110

[50] Xian Tao L, Dyachenko V, Zuzarte M, Putzke C, Preisig-Muller R, et al (2006) The stretch-activated potassium channel TREK-1 in rat cardiac ventricular muscle. Cardiovasc Res 69:86–97. 16248991 10.1016/j.cardiores.2005.08.018

[51] Gruss M, Mathie A, Lieb WR, Franks NP (2004) The two-pore-domain K(+) channels TREK-1 and TASK-3 are differentially modulated by copper and zinc. Mol Pharmacol 66:530–537. 15322244 10.1124/mol.66.3.

[52] Mathie A, Sutton GL, Clarke CE, Veale EL (2006) Zinc and copper: pharmacological probes and endogenous modulators of neuronal excitability. Pharmacol Ther 111:567–583. 16410023 10.1016/j.pharmthera.2005.11.004

[53] Bai X, Greenwood SL, Glazier JD, Baker PN, Sibley CP, et al (2005) Localization of TASK and TREK, two-pore domain K+ channels, in human cytotrophoblast cells. J Soc Gynecol Investig 12:77–83. 10.1016/j.jsgi.2004.08.004 15695101

[54] Divya CS, Pillai MR (2006) Antitumor action of curcumin in human papillomavirus associated cells involves downregulation of viral oncogenes, prevention of NFkB and AP-1 translocation, and modulation of apoptosis. Mol Carcinog 45:320–332. 16526022 10.1002/mc.20170

[55] Aalkjaer C (1990) Regulation of intracellular pH and its role in vascular smooth muscle function. J Hypertens 8:197–206. 2159500 10.1097/00004872-199003000-00001

[56] Ramsey IS, Delling M, Clapham DE (2006) An introduction to TRP channels. Annu Rev Physiol 68:619–647. 16460286 10.1146/annurev.physiol.68.040204.100431

[57] Ashworth JR, Baker PN, Warren AY, Johnson IR (1999) Mechanisms of endothelium-dependent relaxation in myometrial resistance vessels and their alteration in preeclampsia. Hypertens Pregnancy 18:57–71. 10464000 10.3109/10641959909009611

[58] Yang JM, Wang KG (1995) Relationship between acute fetal distress and maternal-placental-fetal circulations in severe preeclampsia. Acta Obstet Gynecol Scand 74:419–424. 7604683 10.3109/00016349509024402

[59] Roberts JM, Cooper DW (2001) Pathogenesis and genetics of pre-eclampsia. Lancet 357:53–56. 11197372 10.1016/s0140-6736(00)03577-7

[60] Hubel CA (1999) Oxidative stress in the pathogenesis of preeclampsia. Proc Soc Exp Biol Med 222:222–235. 10601881 10.1177/153537029922200305

[61] Mills TA, Wareing M, Shennan AH, Poston L, Baker PN, et al (2009) Acute and chronic modulation of placental chorionic plate artery reactivity by reactive oxygen species. Free Radic Biol Med 47:159–166. 19389471 10.1016/j.freeradbiomed.2009.04.019

[62] Allen J, Maigaard S, Christensen JH, Andreasen F, Forman A (1987) Effects of thiopentone or chlormethiazole on human placental stem villous arteries. Br J Anaesth 59:1273–1277. 3479154 10.1093/bja/59.10.1273

[63] Bayliss DA, Barrett PQ (2008) Emerging roles for two-pore-domain potassium channels and their potential therapeutic impact. Trends Pharmacol Sci 29:566–575. 18823665 10.1016/j.tips.2008.07.013 PMC2777628

